# Application of ddPCR in detection of the status and abundance of EGFR T790M mutation in the plasma samples of non-small cell lung cancer patients

**DOI:** 10.3389/fonc.2022.942123

**Published:** 2023-01-26

**Authors:** Hui Zhang, Yi Hu, Yan Wang, Xia Song, Ying Hu, Li Ma, Xinjie Yang, Kun Li, Na Qin, Jinghui Wang, Jialin Lv, Xi Li, Xinyong Zhang, Quan Zhang, Yuhua Wu, Guangyin Yao, Shucai Zhang

**Affiliations:** ^1^ Department of Medical Oncology, Beijing Chest Hospital, Capital Medical University/Beijing Tuberculosis and Thoracic Tumor Research Institute, Beijing, China; ^2^ Department of Medical Oncology, Chinese People's Liberation Army General Hospital, Beijing, China; ^3^ Department of Medical Oncology, National Cancer Center/National Clinical Research Center for Cancer/Cancer Hospital, Chinese Academy of Medical Sciences and Peking Union Medical College, Beijing, China; ^4^ Department of Respiratory, Shanxi Cancer Hospital, Affiliated Cancer Hospital of Shanxi Medical University, Taiyuan, China; ^5^ Department of Pathology, Beijing Chest Hospital, Capital Medical University/Beijing Tuberculosis and Thoracic Tumor Research Institute, Beijing, China; ^6^ Department of Medicine, Shanghai Yuanqi Biomedical Technology Co. Ltd., Shanghai, China

**Keywords:** *EGFR* T790M, ddPCR, NGS, plasma, third-generation EGFR-TKIs

## Abstract

**Background/Objective:**

The third-generation epidermal growth factor receptor (*EGFR*) -tyrosine kinase inhibitor (TKIs), such as osimertinib, designed for targeting the acquired drug-resistant mutation of *EGFR* T790M, was approved as the first-line therapy for advanced *EGFR*-mutated non-small cell lung cancer (NSCLC). Thus, detection of the *EGFR* T790M mutation for NSCLC is crucial. However, tissue samples are often difficult to obtain, especially in patients at advanced stages. This study assessed the performances of droplet digital polymerase chain reaction (ddPCR) and next-generation sequencing (NGS) in detecting *EGFR* T790M status and abundance in the plasma ctDNA samples of patients with NSCLC. We also explored the association between T790M status and abundance and the response to third-generation EGFR-TKIs.

**Methods:**

A total of 201 plasma samples with matched tissues, 821 plasma samples, and 56 patients who received third-generation EGFR-TKIs with response evaluation were included in this study. ddPCR and NGS were used to detect the mutation status and abundance of T790M in the tissues and/or blood samples.

**Results:**

The results showed that the sensitivity and the specificity of *EGFR* T790M mutation status detected by ddPCR in plasma samples were 81.82% and 91.85%, respectively, compared with the tissue samples, with a consistency coefficient of 0.740. Among the 821 plasma samples, the positive rates of *EGFR* T790M detected by ddPCR and NGS were 34.2% (281/821) and 22.5% (185/821), respectively. With NGS results as the reference, the sensitivity and the specificity of ddPCR were 100% and 84.91%, respectively, and the consistency coefficient of the two methods was 0.717. In addition, we found that a higher *EGFR* T790M abundance was linked to a higher treatment response rate to the third-generation EGFR-TKIs regardless of the classification of the median value of 0.43% (*P* = 0.016) or average value of 3.16% (*P* = 0.010).

**Conclusion:**

Taking these data together, this study reveals that ddPCR is an alternatively potent method for the detection of *EGFR* T790M in the plasma samples of NSCLC patients.

## Introduction

P.Thr790Met (T790M) mutation of the epidermal growth factor receptor (*EGFR*) occurs in approximately 50%–60% of non-small cell lung cancer (NSCLC) patients who received first-generation EGFR-TKIs (tyrosine kinase inhibitors) and is one of the main mechanisms of acquired drug resistance (1–3). Fortunately, introduction of the third-generation EGFR-TKIs, such as osimertinib which targets the T790M mutation, provides a powerful treatment option (4–6). Osimertinib can potently inhibit both EGFR-TKI-sensitizing mutations (*EGFR*m) and *EGFR* T790M mutation and has been approved as the first-line therapy for advanced NSCLC patients with *EGFR* mutation in 2018 (4–6). Thus, detection of the *EGFR* T790M mutation in NSCLC patients is crucial for treatment choice.

NSCLC patients with advanced stage and acquired resistance to TKIs are usually not candidates for surgical resection, thus challenging *EGFR* T790M mutation detection (7–9). Therefore, the development of accurate liquid biopsy to determine the status and abundance of T790M mutation is extremely urgent (10–12), as tumor DNA can be obtained either from tissue biopsy gDNA or plasma-circulating tumor DNA (ctDNA). Plasma ctDNA mutation testing has distinct advantages over tumor tissue biopsy such as low cost, decreased risk of complication, and shorter turnaround time (10). Currently, next-generation sequencing (NGS) and droplet digital polymerase chain reaction (ddPCR) are two effective methods to detect ctDNA mutations. In detail, NGS is the exclusive method that can be used for the simultaneous detection of multiple genes or even the whole genome and is of great importance in the era of individualized treatment. However, the cost of NGS is high, and its sensitivity can only reach 10^-2^ generally, or it could be increased to 10^-3^ with a higher cost. On the contrary, ddPCR is a high-sensitivity assay which can rapidly and accurately detect single or several mutation sites including *EGFR* T790M in formalin-fixed, paraffin-embedded (FFPE) tissues and plasma ctDNA with less cost. In clinical detection, ddPCR has been used for T790M mutation detection (13, 14).

In this study, we compared the performance of ddPCR and NGS in detection of the status and abundance of *EGFR* T790M mutation in plasma ctDNA samples. Furthermore, we assessed the correlation between plasma T790M mutation abundance and the clinical response to third-generation EGFR-TKIs, as well as the prognosis of patients with NSCLC.

## Materials and methods

### Plasma and tumor sample collection

Plasma and tissue samples were collected from patients with lung adenocarcinoma from Beijing Chest Hospital, Cancer Chinese PLA (People's Liberation Army) General Hospital, Hospital Chinese Academy of Medical Sciences and Shanxi Provincial Cancer Hospital between December 2009 and April 2019 (**Table 1**). All samples were obtained before the application of third-generation EGFR-TKIs, with an interval of less than 21 days between the tissue and matched plasma samples. In total, 821 plasma samples were collected for T790M mutation detection by ddPCR and NGS, and 201 matched tissue and plasma samples were used for T790M mutation detection by ddPCR. Plasma ctDNA was extracted by QIAamp Circulating Nucleic Acid kit (Qiagen, Hilden, Germany) based on the manufacturer’s instructions. Tissue gDNA was extracted from FFPE or fresh tumor samples by QIAamp FFPE tissue kit (Qiagen, Hilden, Germany). T790M mutation was detected by Human *EGFR* Gene T790M Mutation Detection kit (ddPCR) (Yuanqi Biomedical Technology Co., LTD., Shanghai, China) or Illumina MiniSeq instrument (NGS) according to the manufacturer’s instructions.

**Table 1:** Sample sources.

Hospital	Plasmas	Paired tissues	Total
Beijing Chest Hospital	94	75	169
Chinese PLA General Hospital	200	126	326
Cancer Hospital Chinese Academy of Medical Sciences	207	/	207
Shanxi Provincial Cancer Hospital	119	/	119
Total	620	201	821

**Figure 1:**
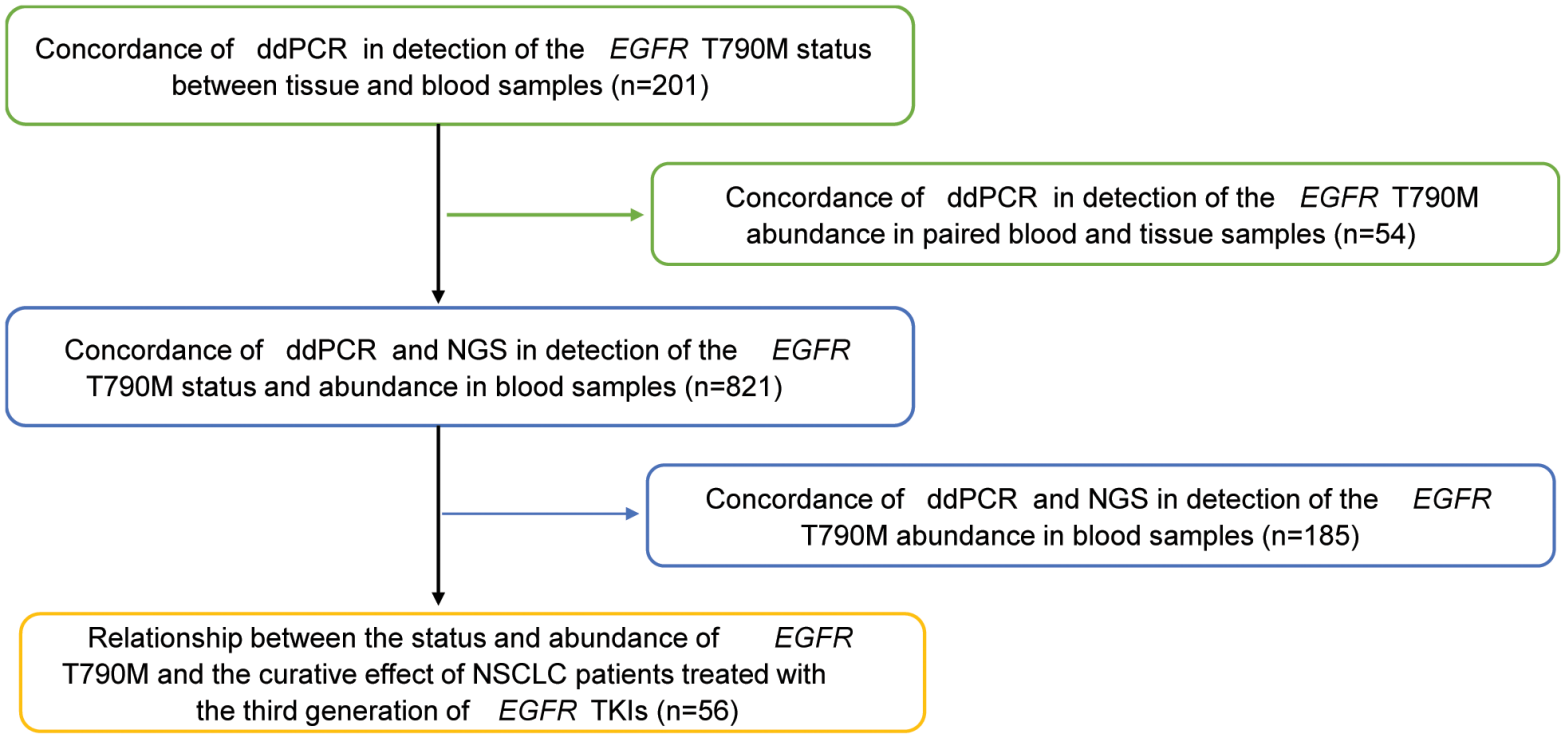
Flow chart of this study.

In addition, a total of 56 patients with lung adenocarcinoma in Beijing Chest Hospital from December 2009 to April 2019 were analyzed retrospectively to assess the relationship between T790M mutation status/abundance and the response to third-generation EGFR-TKIs, as well as the prognosis. All patients were pathologically diagnosed with advanced stage (IV) NSCLC and acquired resistance to first/second-generation EGFR-TKIs (gefitinib, erlotinib, or icotinib) and then given the third-generation EGFR-TKIs, including osimertinib or alflutinib, orally at a recommended dose. The acquired resistance to EGFR-TKIs was evaluated based on Jackman’s clinical definition. During the treatment period, a regular imaging review was conducted to track the progress of the disease. Clinical information for the 56 patients treated with the third-generation EGFR-TKIs was collected for further statistical analysis. **Figure 1** shows the assessment flow chart of this study.

### Response evaluation

Objective tumor responses were evaluated every 6–8 weeks in accordance with the Response Evaluation Criteria in Solid Tumors guidelines (version 1.1) with the following definitions: complete response (CR), partial response (PR), stable disease (SD), and disease progression (PD).

### Statistical analysis

SPSS 23.0 (IBM Corp., Armonk, NY, USA) and GraphPad Prism 9 (La Jolla, CA, USA) software were used for statistical analyses and graphical representations. Mann–Whitney *U*-test was applied to compare the abundance of *EGFR* T790M mutation in patients who achieved CR+PR and SD+PD. Chi-square and Fisher’s exact tests were used to analyze the correlations between clinicopathological features and the response to third-generation EGFR-TKIs. Paired *t*-test and Bland–Altman analysis were used to assess the consistency of the test results from two detection methods. Kaplan–Meier curves with log-rank tests were used to assess the relationship between *EGFR* T790M abundance and the overall survival (OS) of patients with NSCLC. All statistical tests were two-sided, and *P <*0.05 was considered as statistically different.

## Results

### Concordance of the status and abundance of *EGFR* T790M mutation between plasma and tissue samples by ddPCR

**Figure 2:**
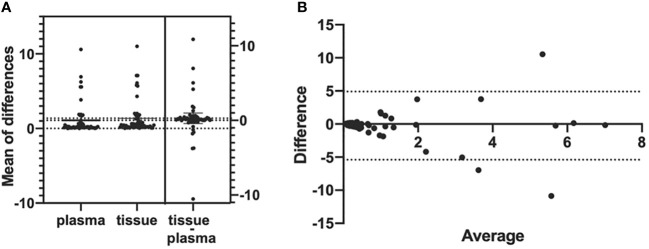
Bland–Altman agreement plots of T790M abundance detected in plasma and tumor tissues by ddPCR. **(A)** Mean of the differences of 54 T790M-positive samples’ mutation abundance in both plasma samples and tumor tissue samples; *P*-value >0.05, no significance. **(B)** The Bland–Altman bias of differences between plasma samples and tumor tissue samples detected *EGFR* T790M abundance.

A total of 201 paired plasma and tissue samples were enrolled in this analysis. The *EGFR* T790M detection rate by ddPCR assay was 32.3% (65/201) in plasma samples and 32.8% (66/201) in the paired tumor tissue samples. With tumor tissue results as the reference, the sensitivity and the specificity for *EGFR* T790M mutation in plasma samples were 81.82% and 91.85%, respectively (**Table 2**). The consistency coefficient of *EGFR* T790M in paired plasma and tissue samples detected by ddPCR was 0.740 (0.6 ≤ Kappa < 0.85, *P* < 0.001). We also assessed the concordance of ddPCR in the detection of the abundance of *EGFR* T790M in 54 paired plasma and tissue samples which were T790M-positive. The paired *t*-test showed that the *P*-value was >0.05 (*P* = 0.4375) (**Figure 2A**). The Bland–Altman bias was -0.28 ± 2.6%, with 95% limits of agreement between -5.4% and 4.9% (**Figure 2B**). These results suggested a high consistency of ddPCR in the detection of the status and abundance of *EGFR* T790M in plasma and tissue samples.

**Table 2:** Detection of *EGFR* T790M status in 201 paired plasma ctDNA and tumor tissues by ddPCR.

		ddPCR-T790M (tissue)			Total
		Negative	Positive		
ddPCR-T790M (plasma)	Negative	124	12	136	
	Positive	11	54	65	
	Total	135	66	201	

### Concordance between ddPCR and NGS in detection of the status and abundance of plasma *EGFR* T790M mutation

**Figure 3:**
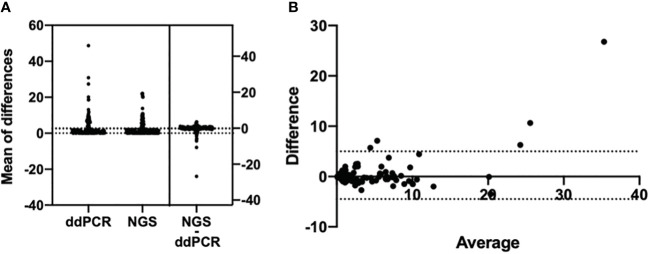
Bland–Altman agreement plots of T790M mutation abundance detected by ddPCR assay and NGS. **(A)** Mean of the differences of 185 T790M-positive samples’ mutation abundance in both the ddPCR assay and the NGS assay; *P*-value >0.05, no significance. **(B)** Bland–Altman bias of differences between ddPCR assay and NGS assays detected *EGFR* T790M abundance.

A total of 821 plasma samples were enrolled in this analysis. The positive rates of *EGFR* T790M mutation detected by ddPCR and NGS were 34.2% (281/821) and 22.5% (185/821), respectively. With NGS results as the reference, the sensitivity and the specificity for ddPCR were 100% and 84.91%, respectively (**Table 3**). The consistency coefficient of the two methods was 0.717 (0.6 ≤ Kappa < 0.85, *P* < 0.001). We also assessed the concordance of ddPCR and NGS in the detection of the abundance of plasma T790M mutation in 185 samples which were positive for T790M mutation as detected by both ddPCR and NGS. The paired *t*-test showed that the *P*-value was >0.05 (*P* = 0.1233) (**Figure 3A**). The Bland–Altman bias was 0.28 ± 2.4% with 95% limits of agreement between -4.5% and 5.0% (**Figure 3B**), suggesting that there was no significant difference between the ddPCR and NGS assays in detecting *EGFR* T790M mutation abundance.

**Table 3:** Consistency between ddPCR and NGS in the detection of plasma *EGFR* T790M status in 821 samples.

		NGS-T790M status		Total
		Negative	Positive	
ddPCR-T790M status	Negative	540	0	540
	Positive	96	185	281
	Total	636	185	821

**Figure 4:**
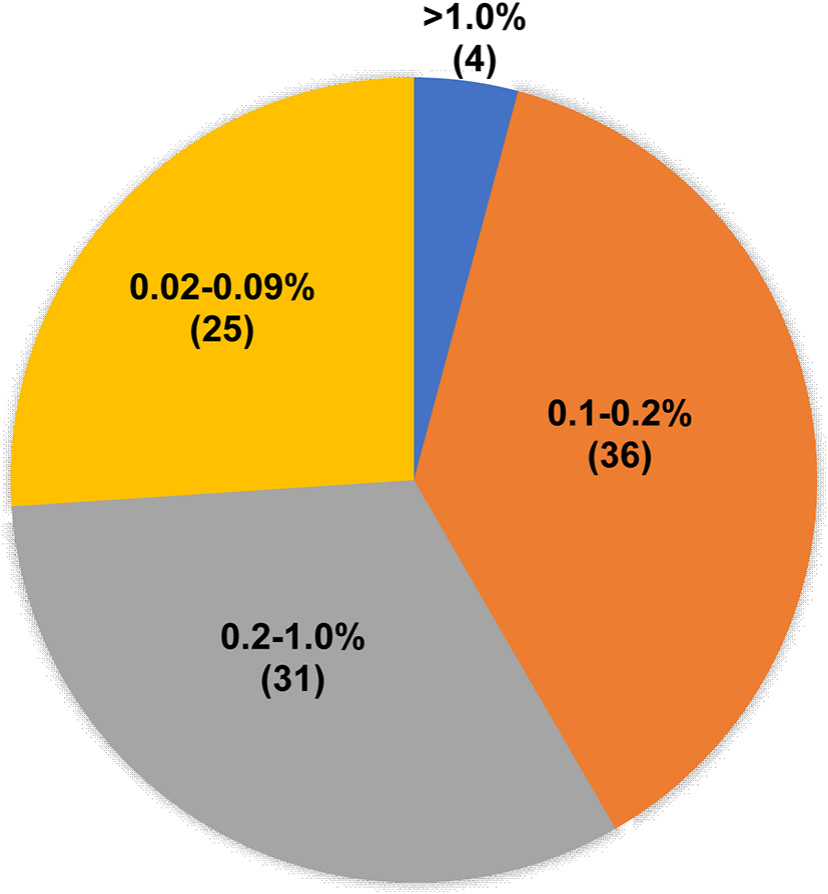
T790M mutation abundance of ddPCR T790M-positive but NGS T790M-negative samples. Pie chart showing the T790M abundance distribution of 96 ddPCR T790M-positive but NGS T790M-negative samples.

In addition, we analyzed the abundance distribution of T790M mutation in 96 samples for which the T790M mutation was positive as detected by ddPCR but negative as detected by NGS. As shown in **Figure 4**, the T790M mutation abundance for 25 samples ranged from 0.02% to 0.09%, which was out of the NGS-positive criteria; for 36 samples it ranged from 0.1% to 0.2%, and for 31 samples it ranged from 0.2% to 1.0%. The abundance of T790M mutation in only four samples was higher than 1.0%.

### *EGFR* T790M mutation abundance was associated with response to third-generation *EGFR-*TKIs and prognosis of NSCLC patients

**Figure 5:**
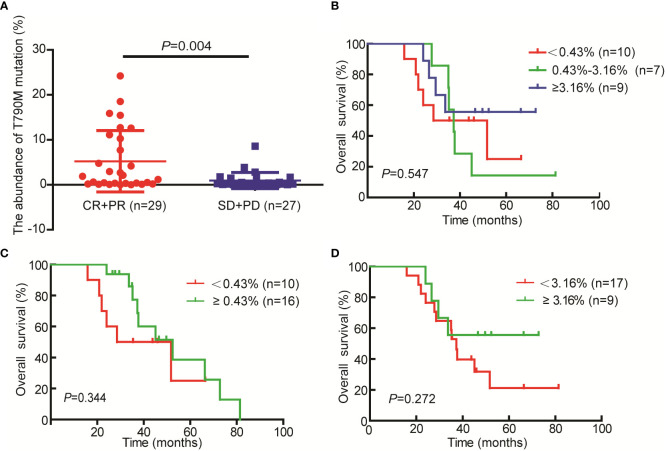
Relationship between T790M mutation abundances and the response to third-generation *EGFR*-TKI and the prognosis of non-small cell lung cancer (NSCLC) patients. **(A)** T790M mutation abundance in the CR+PR and SD+PD groups. **(B–D)** Kaplan–Meier curves were used to assess the relationship between T790M mutation abundance and the overall survival of patients with NSCLC.

A total of 56 NSCLC patients treated with third-generation EGFR-TKIs and who had response evaluation were included in this study. Among the 56 patients, 27 patients (48.2%) were >60 years old, 23 patients (41.1%) were male individuals, 31 patients (55.4%) received alflutinib, and 24 patients (42.9%) received osimertinib. In addition, 29 patients (51.8) achieved PR, 25 patients (44.6%) achieved SD, and two patients (3.6%) achieved PD (**Table 4**). We assessed the relationship between *EGFR* T790M mutation and the response to third-generation EGFR-TKIs in NSCLC patients. The results showed that the *EGFR* T790M mutation status showed no significant association with the response to third-generation EGFR-TKIs in NSCLC patients (*P* = 0.137, **Table 5**). However, we observed that the abundance of *EGFR* T790M mutation was significantly higher in patients who achieved CR and PR compared with that in patients who achieved SD and PD (**Figure 5A**). In addition, a higher *EGFR* T790M mutation abundance was linked to a higher treatment response rate according to the classification of both median (0.43%; *P* = 0.016, **Table 6**) and average (3.16%; *P* = 0.010, **Table 7**). These results indicated that the response rate of NSCLC patients to third-generation EGFR-TKIs was positively related to the abundance of *EGFR* T790M mutation.

**Table 4:** Clinical features of the 56 patients treated with the third generation of *EGFR* tyrosine kinase inhibitors (TKIs).

Clinical features	*n* (%)
Age	
>60	27 (48.2)
≤60	29 (51.8)
Gender	
Male	23 (41.1)
Female	33 (58.9)
First use of third-generation *EGFR* TKIs	
Alflutinib	31 (55.4)
Osimertinib	24 (42.9)
Unknown	1 (1.8)
Curative effective	
PR	29 (51.8)
SD	25 (44.6)
PD	2 (3.6)

**Table 5:** Association between the status of *EGFR* T790M and the curative effect of the third generation of *EGFR* tyrosine kinase inhibitors.

Group	Response (CR+PR), *n* (%)	Non-response (SD+PD), *n* (%)
T790M negative	2 (25)	6 (75)
T790M positive	27 (56.3)	21 (43.7)
*P*	0.137	

**Table 6:** Association between the abundance of *EGFR* T790M (median) and the curative effect of the third generation of *EGFR* tyrosine kinase inhibitors.

Group	≥median (0.43%), *n* (%)	<median (0.43%), *n* (%)
Response (CR+PR)	19 (65.5)	10 (34.5)
Non-response (SD+PD)	9 (33.3)	18 (66.7)
*P*	0.016	

**Table 7:** Association between the abundance of *EGFR* T790M (average) and the curative effective of the third generation of *EGFR* tyrosine kinase inhibitors.

Group	≥average (3.16%), *n* (%)	<median (3.16%), *n* (%)
Response (CR+PR)	11 (37.9)	18 (62.1)
Non-response (SD+PD)	2 (7.4)	25 (92.6)
*P*	0.010	

In addition, we analyzed the relationship between *EGFR* T790M abundance (median and average) and the prognosis of patients. Regrettably, we only got the OS data for 26 cases of the 56 patients. The results demonstrated that patients with higher EGFR T790M abundance especially ≥3.16% (average) had a better prognosis as compared with the patients with lower EGFR T790M abundance (**Figure 5B-5D**). However, the difference doesn’t reach significant, and the small sample size (n=26) may be a possible cause of it.

## Discussion

Clinically, *EGFR* T790M mutation detected in tumor tissues is a gold standard, but it is difficult to obtain tissue samples at some times. With the development of technologies, NGS and ddPCR provide alternative options for the detection of *EGFR* T790M mutation in plasma samples. NGS has advantages of simultaneous detection of multiple genes or even the whole genome with a sensitivity of 0.1%, but its cost is high (15, 16). ddPCR has the advantages of easier set‐up process and faster turnaround time and does not require complex informatics supporting for analysis, with a higher sensitivity of 0.001% (17). However, only known genetic changes can be detected by ddPCR. Currently, the method used to detect *EGFR* T790M mutation in plasma is controversial (18–20). In this study, we compared the performance of ddPCR and NGS in detecting *EGFR* T790M mutation status and abundance in the plasma samples of NSCLC patients. Our results showed that ddPCR is an alternatively potent method for detection of *EGFR* T790M mutation in the plasma samples of NSCLC patients with high sensitivity, lower cost, and shorter experimental period.

Mutation detection using the plasma ctDNA samples shows advantages over the tissue samples, such as easy sample acquisition, little cost, and decreased risks of complications (10). Noticeably, *EGFR*-activating mutations detected using ctDNA samples have been approved as a companion marker to select NSCLC patients for the treatment of osimertinib in the European Union. Nowadays, detection of the *EGFR* T790M mutation in plasma samples has become the research hotspot (21–29)—for example, Krug et al. (30) reported that the sensitivity of T790M mutation detected using plasma ctDNA samples by BEAMing was 82% compared with tissue samples. Papadimitrakopoulou et al. (31) demonstrated that the plasma T790M sensitivity was 58% (110/189) by ddPCR and 66% (136/207) by NGS with cobas^®^ tissue test results as the reference. DNA from tumor cells released into plasma is always accompanied by normal cell-free DNA, leading to ctDNA accounting for only a small fraction. Thus, it is important to use highly sensitive technologies to detect tumor-specific mutations, and ddPCR is just such a technology with a sensitivity of 10^-5^. Herein we assessed the sensitivity and the specificity of ddPCR in detecting *EGFR* T790M mutation in both plasma and paired tissue samples. The T790M mutation positive rate in tissue samples detected by ddPCR was 32.83% (66/201). Taking the tissue sample results as references, the sensitivity and the specificity for ddPCR in detecting *EGFR* T790M mutation in plasma were 81.82% and 91.85%, respectively. Among the 201 samples, 11 cases that were plasma-positive but tissue-negative were identified, with a false-positive rate of 8.15%. Consistently, Oxnard et al. (11) reported that 18 cases were shown to be T790M-negative in tumor samples but positive in plasma, with a false-positive rate of 12.3%. This may be a result of the heterogeneous presence of resistance mutation as a minor clone. As approved by the Food and Drug Administration (FDA), biopsy can be obviated if the plasma genotype is positive for T790M, while it cannot be fully obviated if the plasma genotype of T790M is negative (11). This approval confirms the clinical value of plasma T790M-positive, which can also be verified by published studies which revealed that the patients who are plasma-positive but tissue-negative for T790M mutation could also benefit from osimertinib and have better prognosis (31–33). Nevertheless, some limitations in detecting *EGFR* mutations in ctDNA samples have been clarified—for example, the quantity and the quality of ctDNA vary widely between patients with different kinds of diseases (34). Specifically, the abundance of ctDNA varies from 0.01% to 67% for patients with various cancers or at different stages (35, 36). Herein 12 cases that were tissue-positive but plasma-negative were found among the 201 samples, with a false-negative rate of 18.18%. The false-negative may be caused by the low concentration of blood ctDNAs as well as the interval between tissue and blood sample obtainment. The false-negative rate is lower than the reported data, which is often >30%, due to the limited levels of ctDNA in the blood of patients with relapsed NSCLC (11, 37). The sensitivity and the specificity were also higher than the first FDA-approved Roche cobas^®^ EGFR Mutation Test v2 for T790M detection in blood samples for which the sensitivity was 58.4% and the specificity was 80.4% (30). Moreover, we found that there was no significant difference between plasma and tissue samples at the abundance of *EGFR* T790M, indicating a high consistency.

In addition, we explored ddPCR and NGS in detecting *EGFR* T790M mutation in the plasma samples of patients with NSCLC. With NGS result as the reference, the sensitivity and the specificity of ddPCR in detecting *EGFR* T790M in the plasma samples were 100% and 84.91%. The two methods showed no significant difference between the *EGFR* T790M abundance results, suggesting a good consistency. ddPCR has a higher sensitivity of 0.001% than NGS (38), and in this study, the positive cutoff of ddPCR was set at 0.01%, and that of NGS was set at 0.1% (15, 16). A total of 96 samples were shown to be ddPCR-positive but NGS-negative among the 821 samples. Among the 96 samples, the T790M abundance in 25 samples was lower than 0.1%, lower than the limit of NGS, while the T790M abundance in 67 samples ranged from 0.1% to 1.0%, and four samples showed more than 1.0%. As a limitation of tracking, we found only one patient whose plasma T790M abundance was lower than 0.1% and who received third-generation EGFR-TKI and had available response and prognosis information. This patient achieved PR following alflutinib treatment and had a survival time of more than 5 years and is alive now. The result further verified that ddPCR had a higher sensitivity than NGS.

To further explore the clinical value of T790M-mutation detected by ddPCR, we explored the relationship between T790M mutation status and abundance and the response of NSCLC patients to third-generation EGFR-TKIs. The results showed that patients who were plasma T790M-positive showed a higher response rate than patients who were T790M-negative as detected by ddPCR, but the difference did not reach statistical significance, which may be caused by the small sample size (*n* = 56). We found that NSCLC patients with a high abundance of *EGFR* T790M mutation were associated with a higher response rate to third-generation EGFR-TKIs. In addition, we found that patients with a higher T790M abundance, especially ≥3.16%, had a better prognosis, although the difference did not reach significance. These results further confirmed the clinical value of ddPCR in the detection of T790M mutation in NSCLC patients treated with third-generation EGFR-TKIs.

In conclusion, this study demonstrates that ddPCR is a better choice for the detection of T790M mutation and abundance. T790M abundance detected in plasma samples using ddPCR has clinical values in predicting the response to third-generation EGFR-TKIs in NSCLC patients; specifically, patients with a higher T790M abundance exceeding 0.43% may have a better response to the third-generation EGFR-TKIs. In the future, we intend to collect more samples to determine the relationship between T790M abundance and the response to third-generation EGFR-TKIs as well as the prognosis of patients with NSCLC. Taken together, this study reveals that ddPCR is an alternative method for the detection of *EGFR* T790M mutation in the blood samples of NSCLC patients.

## Data availability statement

The original contributions presented in the study are included in the article/supplementary materials. Further inquiries can be directed to the corresponding author.

## Ethics statement

The studies involving human participants were reviewed and approved by Ethics Committee of Beijing Chest Hospital (No. 2018-07-04 of (2021)). The patients/participants provided their written informed consent to participate in this study.

## Author contributions

HZ, YiH, YWa, XS, and SZ conceived the study. HZ, SZ, and GY designed the study. YingH, LM, and XY analyzed the data. KL, NQ, and JW collected the data. HZ, XL, and XZ wrote the manuscript. JL, QZ, and YWu corrected and approved the final version of the manuscript. All authors contributed to the article and approved the submitted version.
